# A novel innovation using modified platelet-rich plasma (mPRP) for preventing urethral stricture recurrence in a rabbit model

**DOI:** 10.1038/s41598-026-44732-w

**Published:** 2026-04-29

**Authors:** Paksi Satyagraha, Yuyun Yueniwati P.W, Basuki Bambang Purnomo, Edvin Prawira Negara, Putri Wikie Novianti, Athaya Febriantyo Purnomo

**Affiliations:** 1 Department of Urology, Faculty of Medicine Universitas Brawijaya, Malang, Indonesia; 2 Saiful Anwar General Academic Hospital, Malang, Indonesia; 3 Radiology Department, Faculty Medicine, Universitas Brawijaya-Saiful Anwar General Acedemic Hospital, Malang, Indonesia; 4 Siena Clinical, Siena Sains Medika, Jakarta, Indonesia

**Keywords:** Mprp, Preventing, Stricture recurrence, Diseases, Medical research, Urology

## Abstract

Urethroplasty is the gold standard for urethral stricture but is limited by technical complexity and surgeon expertise. Thus, urethrotomy and dilatation remain common despite high recurrence rates (40–60%) from fibrosis. Platelet-rich plasma (PRP), a regenerative therapy, promotes tissue healing but contains transforming growth factor-β1 (TGF-β1), which may worsen fibrosis. To overcome this, a modified PRP (mPRP) was developed by neutralizing TGF-β1, aiming to enhance healing while reducing fibrosis and recurrence in urethral stricture management. An experimental study was conducted using New Zealand White rabbits (Oryctolagus cuniculus). A urethral stricture model was induced through surgical urethral injury followed by TGF-β injection. Animals were treated with either standard PRP or mPRP. Outcomes were assessed through immunohistochemistry (IHC) and retrograde urethrography. Collagen thickness decreased in the PRP group (16.2 ± 2.4 µm; p < 0.05) and further in the mPRP group (11.38 ± 1.46 µm; p < 0.05). IHC revealed lower collagen type III levels in the mPRP group (3.0 ± 1.0) compared with PRP (6.8 ± 0.83), yielding a higher collagen I:III ratio (1:2.6 vs. 1:1.36). Urethrography demonstrated a wider lumen (2.72 ± 0.14 mm) and shorter stricture length (0.60 ± 0.63 cm) in the mPRP group compared to PRP (2.41 ± 0.10 mm and 2.48 ± 0.16 cm; p < 0.05). mPRP effectively reduces fibrosis and inhibits stricture recurrence, as evidenced by decreased collagen expression and improved urethral lumen dimensions, supporting its potential as an adjunctive therapy for urethral stricture management.

## Introduction

Urethral stricture is a narrowing of the urethral lumen caused by fibrotic scarring in the urethral wall, impairing urinary flow. Scar tissue can occur anywhere from the bladder to the urethral meatus and may follow inflammation, infection, or trauma^1^. Alterations in the extracellular matrix (ECM) of urethral tissue and spongiosum are characteristic pathologies; increased synthesis or decreased degradation of ECM components—predominantly collagen—lead to excessive collagen accumulation and fibrosis^2^. Several cytokines regulate collagen synthesis, with transforming growth factor (TGF)-β1 playing a central role^3^. TGF-β1 governs ECM deposition in physiologic tissue repair and in pathologic fibrosis by stimulating fibroblasts and myofibroblasts to overproduce ECM, establishing TGF-β1 as a profibrotic factor^4^.

Multiple surgical techniques—including dilatation, internal urethrotomy, and urethroplasty (end-to-end, graft, flap, two-stage)—are used to manage urethral stricture^5^. Urethroplasty is the gold standard; however, its technical difficulty and need for specialized expertise limit availability^6^. Consequently, internal urethrotomy and dilatation remain frequently chosen for anterior strictures^7^, despite recurrence rates of 40–60% associated with post-procedure fibrotic remodelling^8^. Platelet-rich plasma (PRP) is an endogenous regenerative therapy with the potential to stimulate and accelerate tissue healing^9,10^. As an autologous blood product enriched in platelets, PRP is widely used across clinical applications, including fibrotic diseases^9,10^. Platelets play a key role in wound healing through hemostasis and the release of cytokines and growth factors. Among the growth factors secreted by platelets that contribute to cellular proliferation and tissue regeneration are vascular endothelial growth factor (VEGF), insulin-like growth factor (IGF), transforming growth factor-β1 (TGF-β1), and fibroblast growth factor (FGF)^9,10^. Increasing attention has also been directed toward the roles of Tumor Necrosis Factor -α (TNF-α) (though still controversial), inducible nitric oxide synthase (iNOS), and collagen in fibrosis, underscoring the need for further research into their contributions in urethral stricture^3,11,12^. Because TGF-β1 within PRP may enhance stricture formation, a modified PRP (mPRP) has been developed by neutralizing TGF-β1 to attenuate these profibrotic effects^3,4,11,12^.

## Methods

### Ethical approval and animal care

This study was conducted in accordance with the ARRIVE guidelines (https://arriveguidelines.org) and approved by the Institutional Animal Care and Use Committee (IACUC) of Brawijaya University (protocol number: Approval No. 172/KEP-UB/2023; October 13, 2023). Male *New Zealand White rabbits* (*Oryctolagus cuniculus*), weighing 350–500 g, were obtained from the Laboratory Animal Management Unit (UPHL). New Zealand White rabbits were obtained and housed in the Bioscience Laboratory of Universitas Brawijaya using individual or battery cages measuring 45 cm × 30 cm × 40 cm. A total of 25 rabbits were used in this study. Each cage was equipped with a plastic feeding container and a drinking bottle. The equipment used included cage-cleaning tools, a bench scale, and labeling materials. Cleaning tools were used for maintaining cage hygiene, while writing instruments were utilized for recording daily weight measurements. Rabbits were fed twice daily, once in the morning between 08:00 and 09:00 and once in the afternoon between 16:00 and 17:00. Commercial pellet feed was provided in a plastic feeder typically used for poultry, whereas vegetable waste was given directly into the cages. The commercial pellets were provided before the vegetable waste, as concentrates are preferably administered in the morning, followed by vegetable waste in the afternoon (Tables 1, 2 and 3).

**Table 1:** Collagen thickness by group.

Group	Mean collagen thickness/10 HPF (µm)	p-value
Normal	10,3 ± 0,73	0,00
Stricture	21,15 ± 1,94	
PRP	16,2 ± 2,4	
mPRP	11,38 ± 1,46

**Table 2:** Collagen type I and III expression and I:III ratio.

Group	Collagen I (expr/10 HPF)	p-value	Collagen III (expr/10 HPF)	p-value	Collagen I: III Ratio	p-value
Normal	6,8 ± 1,3	0,014	3,8 ± 1,3	0.05	1: 3	0009
Stricture	10 ± 1,58		10,8 ± 1,3		1: 0,8	
PRP	5,8 ± 2,94		6,8 ± 0,83		1: 1,26	
mPRP	6,6 ± 1,14		3 ± 1		1: 2,6

**Table 3:** Lumen diameter and stricture length.

Group	Lumen diameter (mm)	Stricture length (cm)	p value (diameter)	p value (stricture)
Normal	2.81 ± 0.68	0.00	0,00	0,000
Stricture	0.61 ± 0.16	4,04 ± 0,87		
PRP	2.41 ± 0.1	2,48 ± 0,16		
mPRP	2.72 ± 0.14	0,6 ± 0,63		

### Experimental design

A urethral stricture model was developed using transforming growth factor-β1 (TGF-β1) injection into the ventral penile urethra. Animals were randomly assigned to four groups: (1) Normal, (2) Stricture, (3) platelet-rich plasma (PRP), and (4) modified platelet-rich plasma (mPRP).

### Preparation of platelet-rich plasma (PRP)

Whole blood (3–5 mL per rabbit) was collected from the auricular vein using a sterile wing needle into tubes containing 3.8% sodium citrate as anticoagulant. The samples were centrifuged at 160 × g (≈ 800 rpm) for 20 min to obtain the plasma fraction containing platelets, leukocytes, and a few erythrocytes. The supernatant was transferred to a sterile tube and centrifuged again at 400 × g (≈ 2,000 rpm) for 15 min. After removing half of the supernatant, the remaining pellet was resuspended in the residual plasma to yield PRP (~ 0.8–1.5 mL from 10 mL of blood). Platelet concentrations were measured using an automated hematology analyzer. Aliquots of PRP were collected for ELISA testing to determine TGF-β1 concentration, which was used to guide subsequent mPRP preparation.

### Preparation of modified platelet-rich plasma (mPRP)^13^

To attenuate profibrotic TGF-β1 signaling, PRP was diluted with phosphate-buffered saline (PBS)^14^ and combined with a TGF-β1–neutralizing antibody (rabbit monoclonal TB21 against rabbit TGF-β1, Invitrogen MA1-21,595). mPRP was analysed using the CUSABIO Rabbit Transforming Growth Factor (TGF-β1) ELISA Kit (Catalogue No. CSB-E07000Rb) and compared with PRP and whole blood, demonstrating minimal TGF-β levels. Based on the ELISA results, 0.3 µg antibody was used to neutralize 0.25 ng TGF-β1. No exogenous activator was added after mixing. The rationale for TGF-β1 neutralization step aimed to reduce fibrotic signaling associated with urethral stricture formation.

### Surgical procedure for urethral stricture induction

**Fig. 1:**
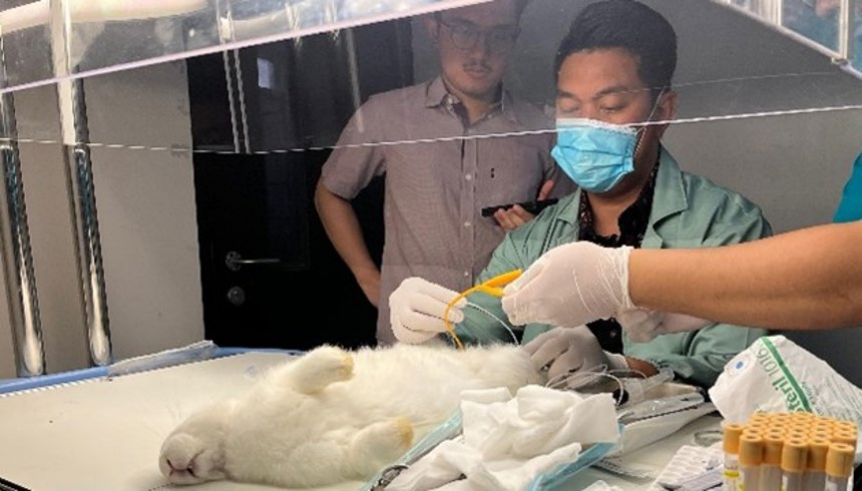
Induction of urethral stricture using TGF-β1 and subsequent PRP/mPRP administration.

Under anesthesia, a 10 Fr catheter pre-marked with methylene blue was inserted into the urethra. A ventral penile skin incision was made to expose the urethral wall. TGF-β1*(*Aviscera Bioscience, Santa Clara, CA*)* in 100 µL saline (6 µg/mL) was injected into the urethral wall (corpus spongiosum) semi-circumferentially along a 1 cm segment at three sites using a 30 G needle. Five minutes later, three punctures were made along the same segment using a 23 G needle until the catheter was visualized (confirmed by methylene blue extravasation), confirming full-thickness penetration. PRP or mPRP was then injected between the TGF-β1 injection sites. The catheter was removed, and the wound was closed using 5–0 non-absorbable monofilament sutures for the deep layer and 5–0 absorbable monofilament sutures for the skin. Clinical observations of prior groups—Sham and TGF-β1 at 1, 2, and 4 µg—were noted at the ventral penile incision line as 1 cm scar markers for localization during imaging. The use of TGF-β1 as a fibrosis trigger and collagen endpoints followed established urethral stricture models. The procedure was performed as described above (Fig. 1).

### Anesthesia and perioperative management

Anesthesia was induced with intramuscular injection of ketamine (20–40 mg/kg) and xylazine (4 mg/kg). For maintenance of anesthesia during the procedure, supplemental intramuscular doses of ketamine (5 mg/kg) and xylazine (1 mg/kg) were administered as needed. Retrograde urethrography (oblique position) was performed during the procedure using a non-ionic contrast agent (150 mg iodine/mL, total volume 10 mL).

### Postoperative evaluation and imaging

At six weeks post-surgery, the ventral penile skin at the previous incision site was inspected. in Sham and TGF-β1 (1, 2, 4 µg) groups the 1 cm scar—demarcated by non-absorbable suture—served as a treatment landmark. The treatment-segment lumen was localized with a needle, and urethrography was obtained. A radiographer, single-blind to group allocation, measured the lumen diameter at the treatment segment and the proximal urethral diameter. These measurements were used to calculate the percentage diameter at the treatment site relative to proximal urethra.

### Euthanasia

Euthanasia was performed using intrathecal administration of 2% lidocaine hydrochloride, in accordance with AVMA Guidelines as a secondary or alternative method in circumstances where barbiturates or other standard agents were unavailable, restricted, or posed disposal challenges. Prior to euthanasia, all animals were maintained under a surgical plane of general anesthesia to ensure unconsciousness. Intrathecal access was obtained by inserting a needle into the subarachnoid space at the lumbosacral region, with correct placement confirmed by the appearance of cerebrospinal fluid (CSF). A sterile 2% lidocaine hydrochloride solution was then administered intrathecally at a dose of approximately 2.6–4 mg/kg body weight via rapid injection. Previous reports have demonstrated that this dose range induces brain death preceding cardiac arrest in horses under intravenous anesthesia, while similar studies in dogs and cats suggest a lethal intrathecal dose of approximately 4–5 mg/kg^11^.

### Histological and immunohistochemical analysis

Tissue samples were collected from the treated urethral segment for histological and immunohistochemical (IHC) analysis. Briefly, the urethral lumen was isolated and fixed in formaldehyde for up to 24 h, followed by paraffin embedding. Tissue sections were obtained from the urethral region where stricture formation was present. Samples underwent dewaxing using dimethylbenzene and were rehydrated through graded ethanol concentrations. Antigen retrieval was performed using a microwave-treated protocol, followed by endogenous peroxidase blocking with H₂O₂. After multiple washes with phosphate-buffered saline (PBS), tissue sections were incubated with primary antibodies at a dilution of 1:100 in distilled water at 4 °C. Following additional PBS washes, peroxidase-labelled secondary antibodies were applied for 15 min at 37 °C. Peroxidase activity was visualised using diaminobenzidine (DAB), resulting in a yellow–brown chromogenic signal. IHC was performed using primary antibodies against collagen type III (ABIN2619397, anti-COL3A1), collagen type I [COL-1] (GTX26308), and ABIN957814 (anti-COL1). Unlabeled primary antibodies (first layer) recognized target antigens in tissue sections, followed by labeled secondary antibodies (second layer) that bind the primary antibodies. Signal development used a chromogenic substrate. Collagen I/III assessments are consistent with established fibrosis readouts in urethral stricture models^12,13^. Histopathological and immunohistochemical analyses were performed by a dedicated anatomical pathologist. Quantitative assessment was assisted by ImageJ software (ImageJ version 1.54 g available at: https://imagej.net/ij/download.html), and all samples were analysed in a double-blinded manner to minimise observer bias.

### Statistical analysis

Data analysis was conducted using SPSS (Statistical Product and Service Solutions) version 25. The obtained data were subjected to distribution and normality tests. Following the assessment of data distribution and normality, the effects of PRP and mPRP on urethral stricture as well as collagen thickness, were evaluated. The effects were analysed using regression analysis.

## Result

**Fig. 2:**
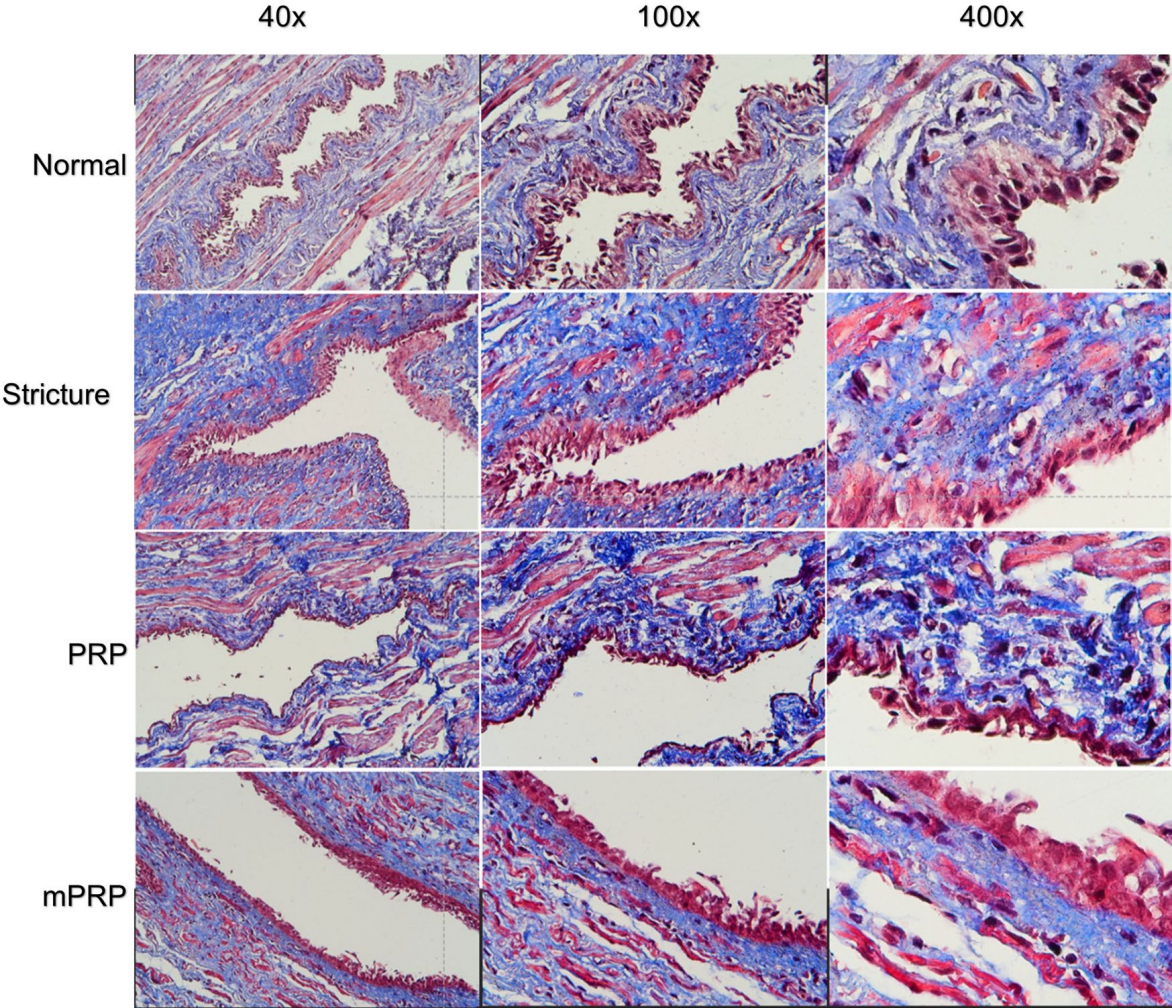
Identification of collagen thickness with Masson’s Trichrome. (**A**) Normal urethra, mean thickness 10.3 ± 0.73 µm; (**B**) Stricture urethra, 21.15 ± 1.94 µm; (**C**) Stricture + PRP, 16.2 ± 2.4 µm; (**D**) Stricture + mPRP, 11.38 ± 1.46 µm.

Mean urethral collagen thickness in the stricture group was 21.15 ± 1.94 µm, whereas the normal group measured 10.30 ± 0.73 µm (Fig. 2); the difference was significant (*p* < 0.05). Treatment reduced collagen thickness in both intervention arms: PRP measured 16.20 ± 2.40 µm and mPRP measured 11.38 ± 1.46 µm; both differed significantly from stricture (p < 0.05), and PRP differed significantly from mPRP (p < 0.05).

**Fig. 3:**
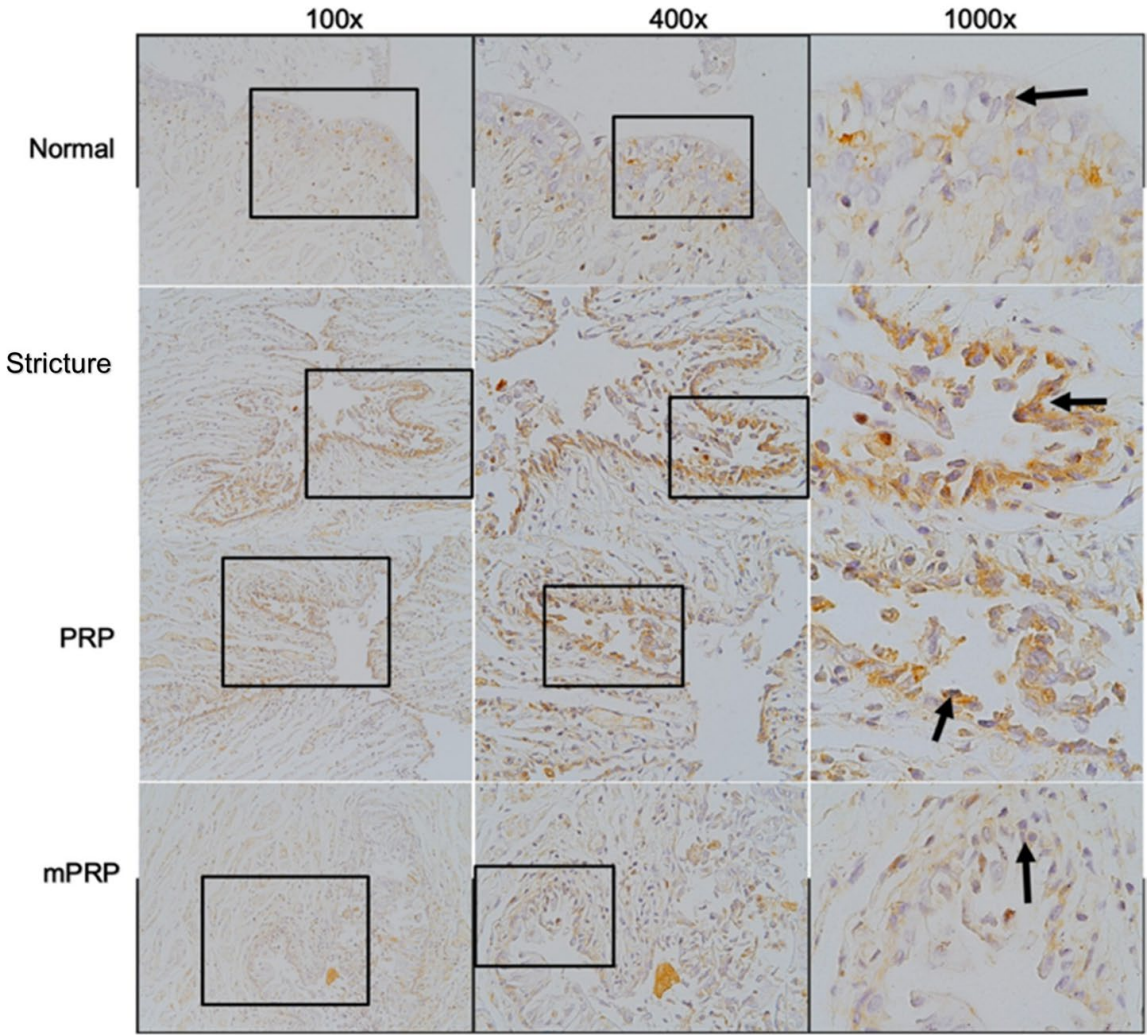
Collagen I expression on immunohistochemical staining. Connective tissue is visualized in brown (black arrows). Collagen I expression in the PRP and mPRP groups appeared similar to that in the normal group.

**Fig. 4:**
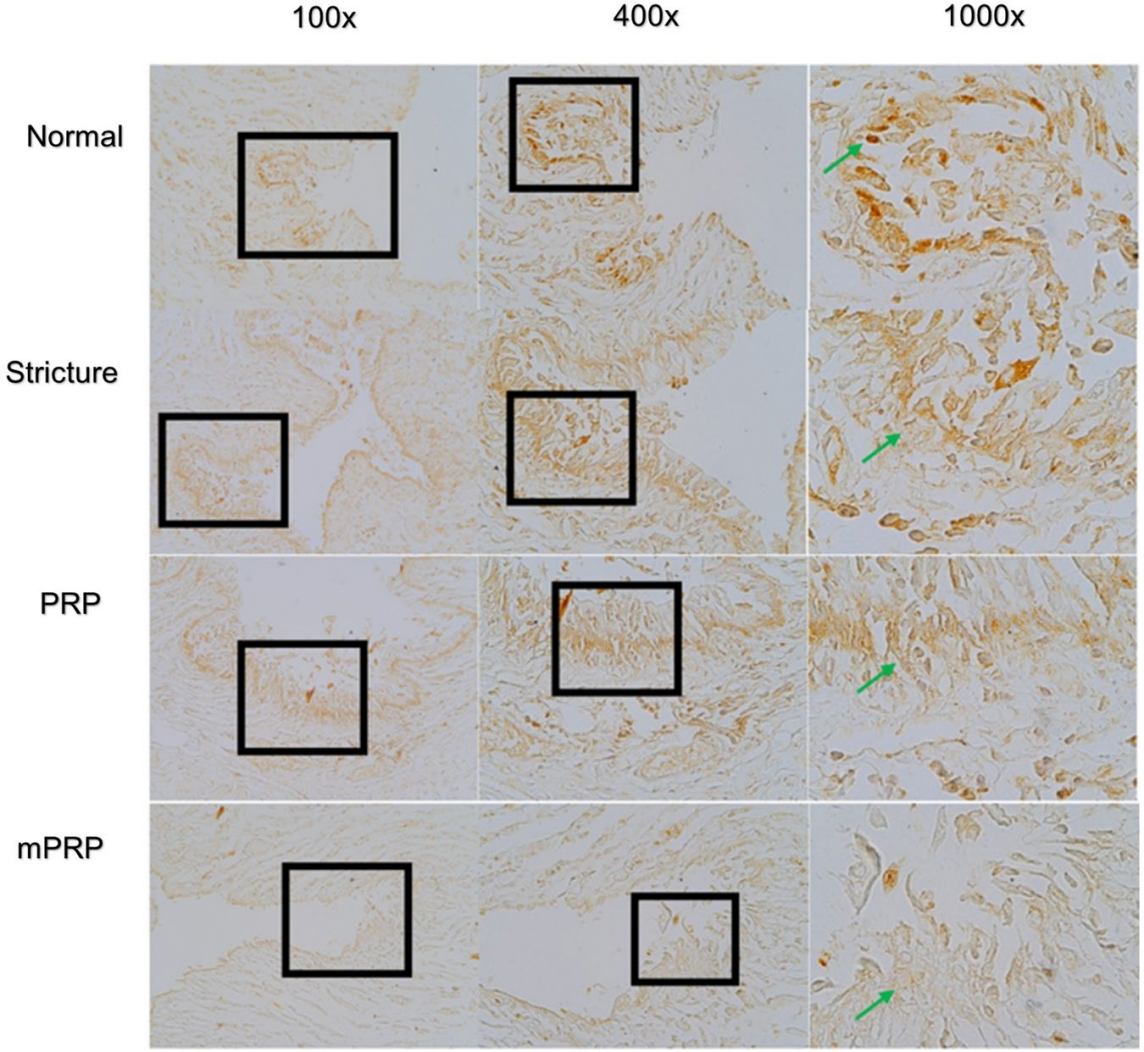
Collagen III expression on immunohistochemical staining, shown as brown coloration in the cytoplasm (black arrows). Collagen III expression in the mPRP group was comparable to the normal group, whereas collagen III expression was lower in mPRP compared with PRP.

On immunohistochemistry (Fig. 3), collagen type I expression (per 10 HPF) was highest in stricture (10.0 ± 1.58), and lower in PRP (5.8 ± 2.94, p < 0.05 vs. stricture) and mPRP (6.6 ± 1.14, p < 0.05 vs. stricture). Collagen type III expression (Fig. 4) was 10.8 ± 1.3 in stricture, 6.8 ± 0.83 in PRP (p < 0.05 vs. stricture), and 3.0 ± 1.0 in mPRP.

**Fig. 5:**
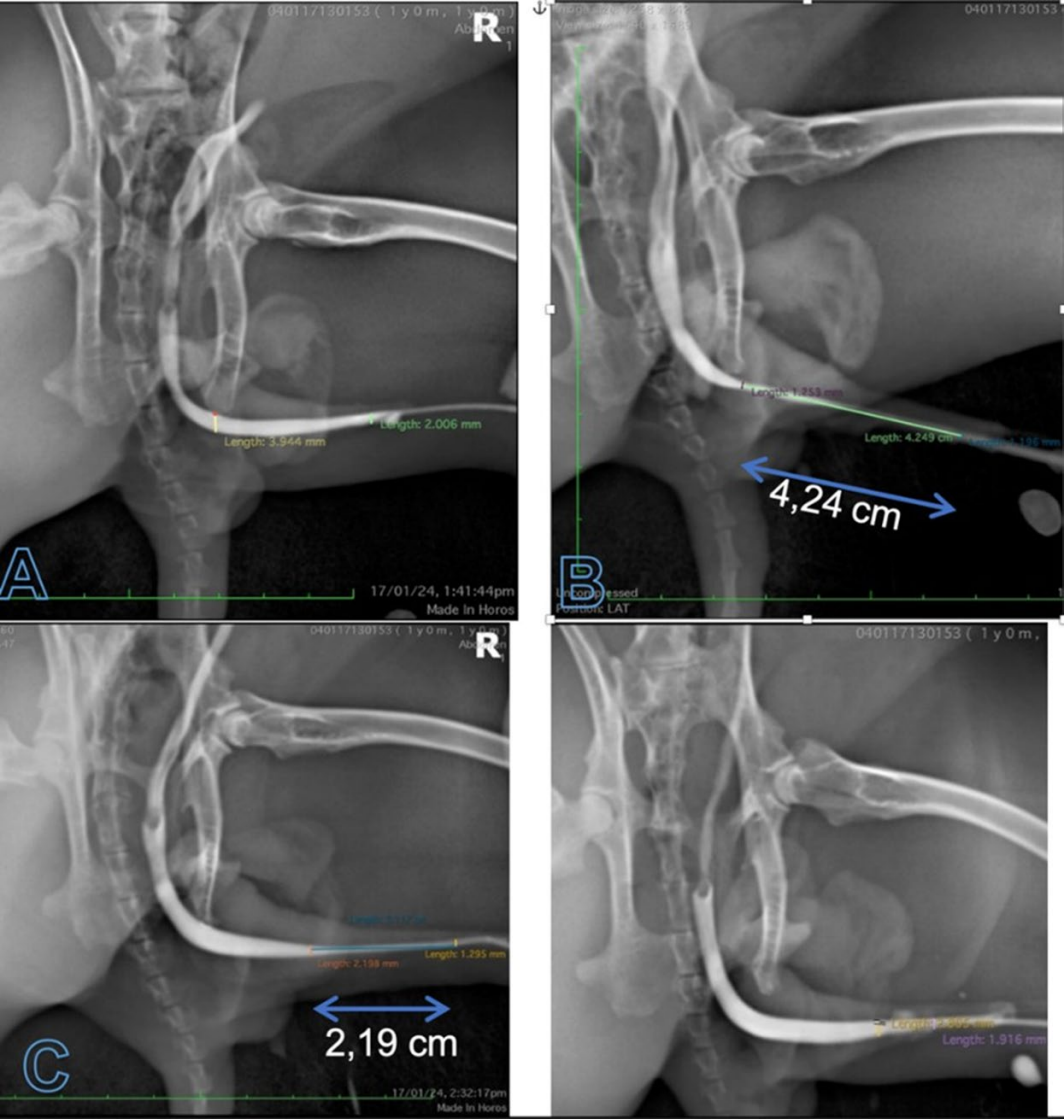
Retrograde urethrography demonstrating urethral stricture formation and treatment response. (**A**) In the normal rabbit, no urethral luminal narrowing was observed. (**B**) In the stricture-induced rabbit, urethral luminal narrowing was evident, with a mean lumen diameter of 0.91 ± 0.16 mm and a mean stricture length of 4.04 ± 0.87 cm. (**C**) In stricture rabbits treated with PRP, the mean urethral lumen diameter increased to 1.32 ± 0.10 mm, with a mean stricture length of 2.48 ± 0.16 cm. (**D**) In stricture rabbits treated with mPRP, further improvement was observed, showing a mean lumen diameter of 1.74 ± 0.14 mm and a mean stricture length of 0.60 ± 0.63 cm.

The collagen I:III ratio—typically ~ 1:3 in normal tissue—was 1:0.8 in stricture. Ratios in treatment groups shifted toward normal: PRP 1:1.26 (*p* > 0.05) and mPRP 1:2.6 (p < 0.05). The normal lumen diameter was 2.81 ± 0.68 mm with no stricture observed. Because no validated radiographic cut-off exists, “stricture” was defined as diameter below the normal mean. Retrograde urethrography demonstrated significant luminal narrowing in the stricture group (Fig. 5). The stricture group showed a lumen diameter of 0.61 ± 0.16 mm and stricture length of 4.04 ± 0.81 cm. Treatment improved both measures: PRP achieved 2.41 ± 0.10 mm lumen and 2.48 ± 0.16 cm stricture length (both *p* < 0.05), while mPRP achieved 2.72 ± 0.14 mm and 0.60 ± 0.63 cm, respectively.

**Fig. 6:**
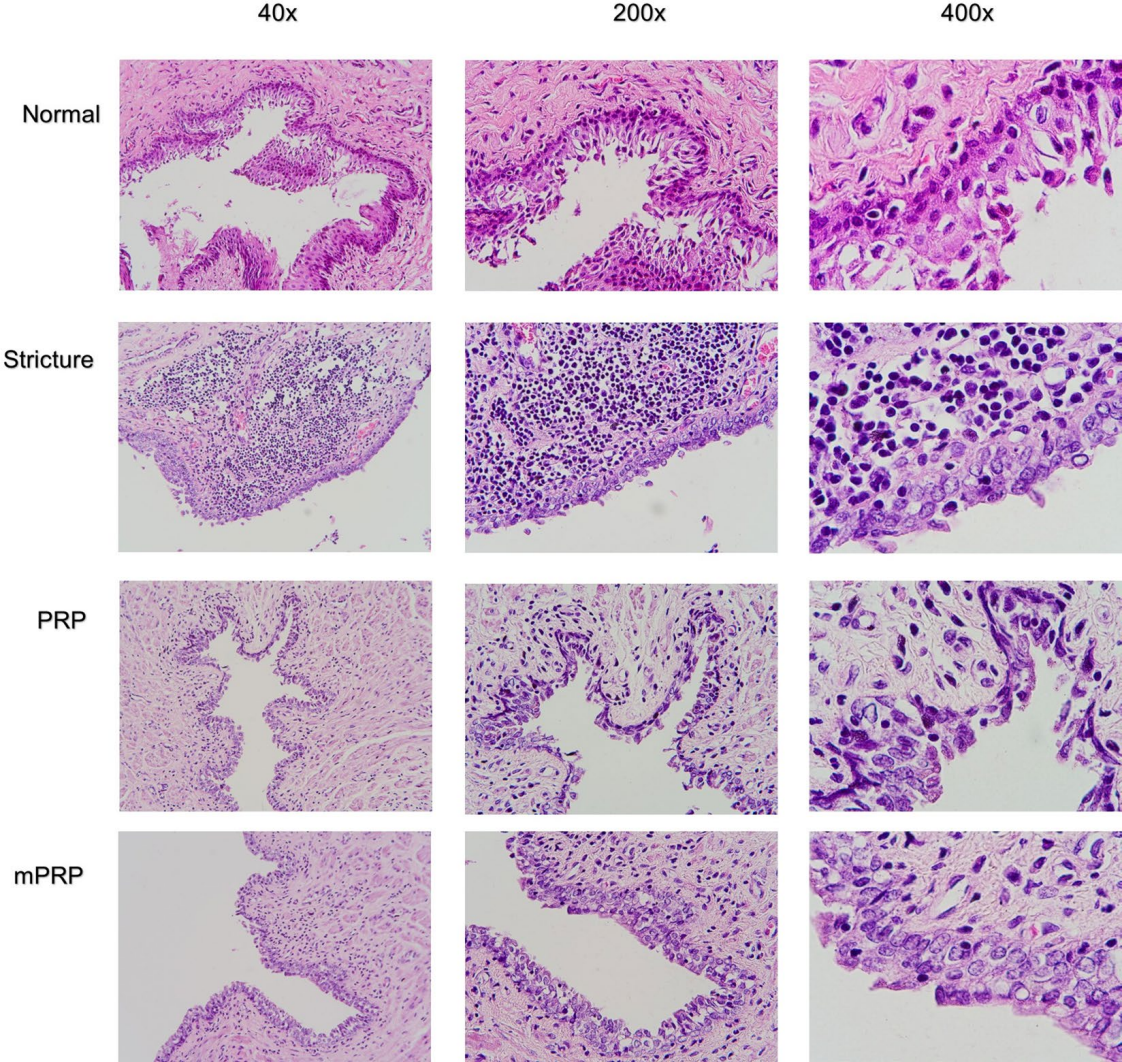
Histological evaluation of urethral stricture formation (H&E staining)**.** Low-magnification images (× 40) demonstrate overall urethral architecture and marked luminal narrowing in the stricture group. Higher-magnification images (× 200 and × 400) reveal increased collagen deposition, subepithelial fibrosis, and inflammatory cell infiltration within the corpus spongiosum, consistent with urethral stricture formation.

Histological analysis further confirmed stricture formation (Fig. 6). Low-magnification images demonstrated marked luminal narrowing and thickening of the urethral wall in the stricture group compared with sham controls. High-magnification views revealed dense subepithelial fibrosis and increased collagen deposition within the corpus spongiosum. Treatment with PRP and mPRP reduced fibrotic changes and partially restored urethral architecture.

## Discussion

After internal urethrotomy, wound healing begins within the incised urethral tissue. Submucosal PRP injection following urethrotomy is intended to enhance healing and reduce stricture recurrence. In our summary of prior reports, submucosal PRP did not differ from control at early follow-up, but by 12 months stricture recurrence was more frequent in controls (26.82%) than in the PRP group (9.09%) (*p* = 0.032), with a protective effect persisting over the subsequent 12 months (*p* = 0.034)^15^. These observations are biologically plausible given PRP’s pro-healing profile in soft-tissue repair and the central profibrotic role of TGF-β1 in urethral fibrosis.

Preclinical and surgical reports support an anti-stricture role for PRP: Tavukcu et al. showed PRP protects against urethral stricture formation and prevents an increase in the collagen type I/III ratio in a rat urethral-injury model^16^; in an uncontrolled series, Scarcia et al. used autologous PRP gel to improve buccal mucosa graft vascularization and reduce fibrosis in bulbar/penile urethroplasty, reporting no recurrences and no major complications^17^. In line with this paradigm, our study found significant histologic differences between the PRP group and the stricture group.

A key rationale for testing a TGF-β1–neutralized, modified PRP (mPRP) is the duality of PRP biology: while PRP accelerates repair, it also contains abundant TGF-β1, a driver of pathological fibrosis and scarring^3^. Variability in PRP composition (e.g., leukocyte content) and target tissue may yield divergent fibrotic outcomes^9,10^. Reviews summarized by Alves & Grimalt indicate that neutralizing TGF-β1 in PRP reduces collagen deposition versus non-customized PRP, supporting the hypothesis that TGF-β1 neutralization attenuates fibrogenesis^10^. Our data align with this mechanism: compared with PRP, mPRP reduced collagen I and III expression toward normal and decreased overall collagen thickness, consistent with dampened TGF-β1 signaling.

With respect to ECM remodeling, we evaluated collagen I, collagen III, and total collagen thickness. Collagen I expression did not differ significantly across groups, suggesting a limited role for type I alone in this model; in contrast, collagen III expression was significantly lowered by mPRP and approached normal. Masson’s Trichrome further showed that total collagen thickness in mPRP was markedly lower than in positive controls and not significantly different from normal, indicating substantial mitigation of fibrotic deposition—findings consistent with known ECM alterations in stricture and TGF-β1-driven collagen dynamics.

Anatomically, retrograde urethrography demonstrated that lumen diameter in the mPRP group was wider than in positive controls and PRP and not significantly different from normal; stricture length was likewise shorter than in positive controls and PRP and not different from normal. Together, imaging corroborates the histology, showing that mPRP restores urethral caliber and limits stricture extent.

Overall, these data address debate around modified PRP for urethral stricture. mPRP preserves PRP’s regenerative benefits while functionally reducing TGF-β1 exposure, thereby lowering ECM accumulation and normalizing the collagen I:III balance. In this model, mPRP was superior to PRP across inflammatory/remodeling pathways and anatomical outcomes, supporting mPRP as a more effective antifibrotic adjunct to prevent recurrence.

This study has several limitations. First, the experiment was performed in a rabbit model, which may not fully replicate the complexity of urethral stricture formation and healing in humans. Second, the sample size was limited, and longer observation periods could better capture the long-term effects of modified platelet-rich plasma (mPRP) on fibrosis and recurrence. Third, only histological and radiographic parameters were evaluated; molecular analyses of fibrotic signalling pathways and inflammatory mediators would further clarify the mechanisms underlying mPRP’s antifibrotic effects.

## Conclusion

Administration of mPRP inhibited the recurrence of urethral stricture by reducing collagen expression, with anatomical confirmation on urethrography. These findings demonstrate that neutralizing TGF-β within PRP can prevent stricture formation at both histologic (physiologic) and anatomic levels.

## Data Availability

De-identified data underlying the findings of this study are available from the corresponding author upon reasonable request. The analysis scripts and study materials are available on request. Authors are willing to deposit datasets in an appropriate public repository upon acceptance.
